# Physicochemical Characterization, Fatty Acid Profile, and In Vitro Antioxidant Capacity of Tumbo (*Passiflora tripartita* var. *mollissima*) Seed Oil Obtained by Expeller Pressing

**DOI:** 10.3390/foods15173092

**Published:** 2026-08-31

**Authors:** Lizbeth Carolina López Arisaca, Carlos Vílchez-Perales

**Affiliations:** Department of Nutrition, Universidad Nacional Agraria La Molina, Av. La Molina s/n, Lima 15024, Peru; cvilchezp@lamolina.edu.pe

**Keywords:** *Passiflora tripartita* var. *mollissima*, seed oil, antioxidant activity, fatty acid profile, phenolic compounds, expeller pressing

## Abstract

This study evaluated the applicability of tumbo (*Passiflora tripartita* var. *mollissima*) seeds for oil extraction by expeller pressing and characterized the resulting oil in terms of physicochemical quality, fatty acid profile, and in vitro antioxidant properties. The nutritional composition of tumbo fruit fractions, including pulp, peel, seeds, and residual seed cake, was also assessed. Tumbo seed oil showed a pressing yield of 16.78% and was characterized by a predominance of polyunsaturated fatty acids, mainly linoleic acid, together with low acid and peroxide values and a high iodine value. The oil also showed measurable in vitro antioxidant capacity in ABTS, DPPH, and FRAP assays, along with relevant total phenolic content (TPC) and total flavonoid content (TFC). These findings provide baseline information on tumbo seed oil as a non-conventional Andean fruit seed oil and suggest potential interest for future technological and food-related studies. Further research is required to evaluate its oxidative stability, safety, detailed bioactive compound profile, and potential biological effects before proposing specific functional or health-related applications.

## 1. Introduction

The family *Passifloraceae* comprises several genera with a worldwide distribution. Among them, *Passiflora* is the largest genus, with approximately 400 species, most of which are climbing plants with ornamental or other economic importance [1]. They are of great commercial value due to their edible fruits, which include sweet granadilla (*P. ligularis Juss.*) and passion fruit (*Passiflora edulis*). In contrast, tumbo (*Passiflora tripartita* var. *mollissima*) remains a less widely consumed and studied Andean fruit. This species has naturally adapted to different climatic conditions and is native to Peru, Colombia, Ecuador, Panama, and Venezuela. In Peru, it is common in the Andean highlands and is known as “tumbo serrano” [2].

Tumbo is characterized by its elongated oval shape, bright orange pulp, and numerous seeds. The fruit has a sweet–sour flavor and is consumed fresh or processed into juices, jams, beverages, and other food products. Tumbo contains bioactive compounds, including ascorbic acid, carotenoids, and other antioxidants, which support its potential relevance as a fruit with functional and antioxidant value [2].

In contrast, the seeds, which are generally discarded during fruit processing, have recently started attracting attention as valuable sources of vegetable oils rich in essential fatty acids, antioxidants, and other bioactives with potential health benefits. Their valorization as food ingredients not only reduces adverse environmental impacts but also aligns with the principles of a circular economy and sustainable use of natural resources [3].

Several methods have been used to extract oil from these seeds. Among them is expeller pressing, a mechanical method that separates oil from the seed matrix by grinding and pressing the seeds with a screw system inside the press. Mechanical pressing is used in several countries, as the equipment is simple, durable, easy to operate and maintain, and adaptable to different oilseed materials. In addition, compared with solvent extraction, it avoids the use of chemicals and allows the recovery of protein-rich seed cakes free from such residues [4]. Therefore, it may represent a more sustainable alternative for obtaining oils from non-conventional seed sources.

However, limited information is available on the compositional, physicochemical, and antioxidant characteristics of tumbo seed oil obtained by expeller pressing. Therefore, this study aimed to characterize the physicochemical quality, nutritional composition, fatty acid profile, total phenolic content (TPC), total flavonoid content (TFC), and in vitro antioxidant properties of tumbo (*Passiflora tripartita* var. *mollissima*) seed oil. This approach provides initial baseline information on this underutilized Andean fruit seed oil and supports future studies on its oxidative stability, safety, and potential food applications.

## 2. Materials and Methods

### 2.1. Raw Material and Seed Conditioning

Tumbo fruits (*Passiflora tripartita* var. *mollissima*) were obtained from the city of Huánuco, Peru, as a single batch. Fruits were selected at a commercial maturity stage based on external appearance and firmness. The raw material was received in boxes and handled carefully to prevent mechanical damage. The total amount of fruit required was weighed, and fruits showing signs of degradation were removed. The selected fruits were washed with water and disinfected by immersion in a 20 ppm sodium hypochlorite solution, followed by rinsing with water.

After disinfection, the peel or epidermis was removed to obtain the whole pulp, and the seeds were separated through a 2 mm mesh sieve. The seeds were then dried in a tray dryer (model DRR200, Reter Industrias Fraclen, Lima, Peru) at 60 °C for approximately 6–8 h to completely remove the remaining pulp adhering to the seed surface. Subsequently, the dried seeds were manually ground with a domestic grinder (Victoria, Medellín, Colombia) until a particle size of approximately 2 mm was obtained. Before pressing, the moisture content of the ground seeds was adjusted to approximately 10% to facilitate adequate flow through the expeller press and reduce the risk of equipment obstruction. The oil obtained from this single batch was used for the physicochemical, fatty acid, phenolic, flavonoid, and antioxidant analyses.

### 2.2. Seed Oil Extraction by Expeller Pressing

**Figure 1:**
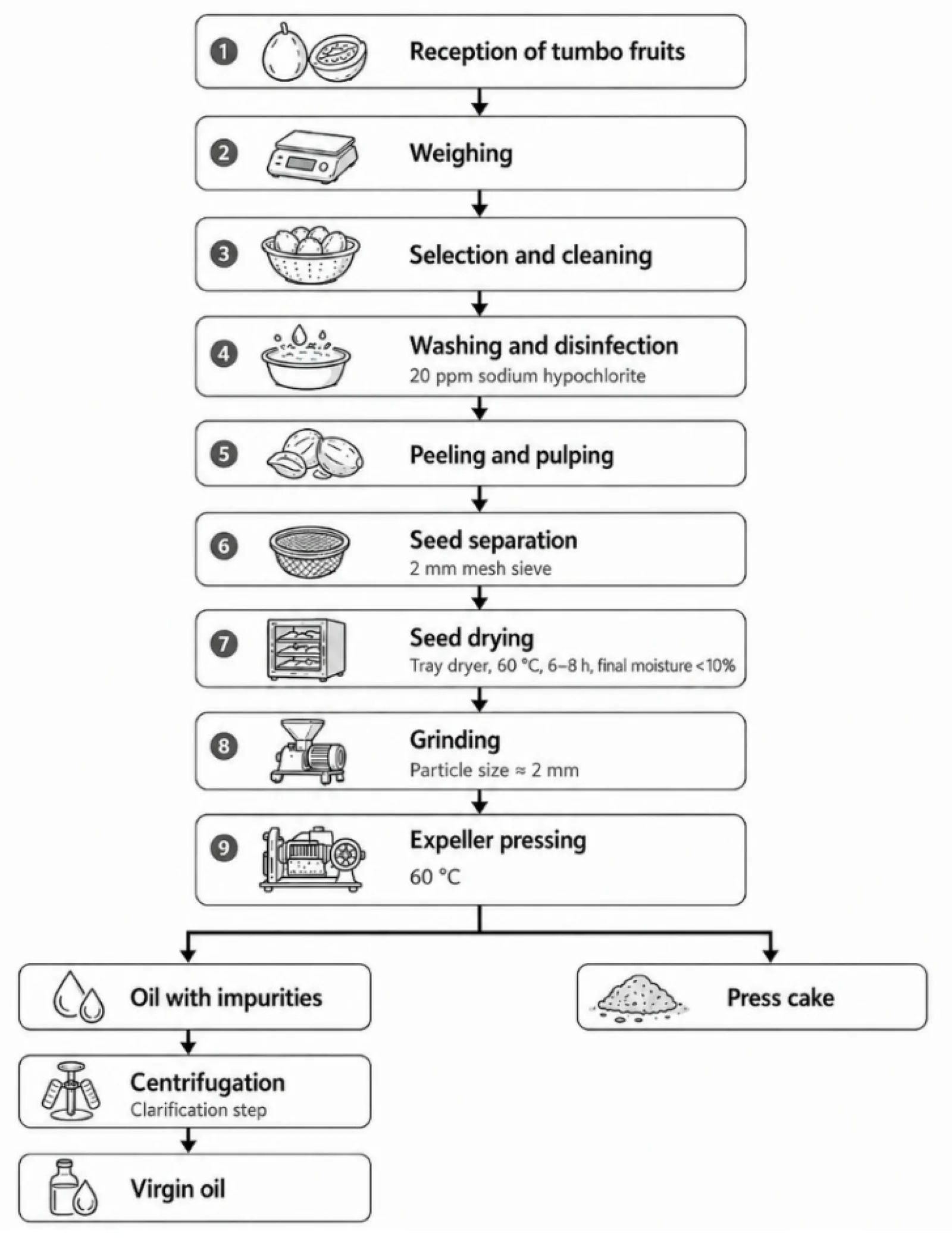
Process flow for obtaining tumbo (*Passiflora tripartita* var. *mollissima*) seed oil by expeller pressing, including raw material conditioning, seed drying, grinding, mechanical pressing, centrifugation, and oil recovery.

The seeds were pressed at 60 °C using a KOMET screw press (CA 59 G; IBG Monforts Oekotec, Mönchengladbach, Germany), at a screw speed of 11 rpm and with a nozzle diameter of 4 mm. Before introducing the seeds into the feed hopper, the press was operated for 15 min with heating through the electric resistance ring fixed around the pressing head, to obtain the selected cylinder temperature [5]. After pressing, the crude oil containing suspended impurities was collected and centrifuged in a laboratory centrifuge (Model 800B; LabTecBio, Lima, Peru) as a clarification step to obtain virgin tumbo seed oil, while the residual press cake was collected as a by-product. The complete process flow is shown in Figure 1.

### 2.3. Oil Pressing Yield

The pressing yield of tumbo seed oil was calculated as the ratio between the weight of the oil obtained and the weight of the processed sample by applying the following equation.

Pressing yield (%) = (Oil weight/Processed seed weight) × 100

### 2.4. Nutritional Composition Analysis

The nutritional composition of tumbo fruit fractions, including pulp, peel, seeds, and residual seed cake, was determined according to the methods prescribed by the Association of Official Analytical Chemists (AOAC) [6]. The analyzed parameters included moisture, crude protein, ether extract, crude fiber, and ash. Nitrogen-free extract was calculated by difference.

### 2.5. Mineral Composition Analysis

The mineral compositions of the tumbo fruit fractions, including pulp, peel, seeds, and residual cake, were determined after wet digestion of the samples, per the AOAC methods [6]. The digested samples were analyzed by atomic absorption spectrophotometry (AAS; PerkinElmer AAnalyst 400, PerkinElmer, Waltham, MA, USA) to quantify calcium (Ca), potassium (K), magnesium (Mg), iron (Fe), zinc (Zn), sulfur (S), sodium (Na), copper (Cu), manganese (Mn), phosphorus (P), nitrogen (N), and boron (B). Results were expressed as percentages (%) for macrominerals and parts per million (ppm) for trace elements.

### 2.6. Fatty Acid Profile

The fatty acid profile of tumbo seed oil was established by gas chromatography–tandem mass spectrometry (GC-MS/MS) using an Agilent 7890B gas chromatograph coupled to an Agilent 7000D triple quadrupole mass spectrometer (Agilent Technologies, Santa Clara, CA, USA), equipped with an Rt™-2560 capillary column (Restek, Bellefonte, PA, USA; biscyanopropyl polysiloxane; 100 m × 0.25 mm ID × 0.20 µm film thickness). The column had maximum and minimum operating temperatures of 250 °C and 220 °C, respectively. Fatty acids were identified using an NLEA FAME MIX standard (cat. no. 35077; Restek, Bellefonte, PA, USA; 30 mg/mL in methylene chloride; batch A054068) containing 28 fatty acid methyl esters. Results were expressed as relative percentages of the total fatty acids detected.

### 2.7. Physicochemical Characterization

The physicochemical characterization of tumbo seed oil included the determination of its iodine, acid, and peroxide values. The iodine value was determined according to International Standard ISO 3961:2018 [7]. The acid value was ascertained following AOCS Official Method Ca 5a-40 [8], and the peroxide value was assessed per AOCS Official Method Cd 8b-90 [9].

### 2.8. In Vitro Antioxidant Capacity

All reagents and standards used in this study were of analytical grade and were purchased from Merck (Darmstadt, Germany). The in vitro antioxidant capacity was evaluated utilizing the 2,2-diphenyl-1-picrylhydrazyl radical-scavenging (DPPH) assay [10], the 2,2′-azino-bis(3-ethylbenzothiazoline-6-sulfonic acid) radical cation decolorization (ABTS) assay [11], and the ferric reducing antioxidant power (FRAP) assay [12]. All determinations were performed in triplicate. Absorbance measurements were performed using a UV–Vis spectrophotometer (Thermo Scientific GENESYS 10S UV–Vis, Thermo Fisher Scientific, Waltham, MA, USA). Trolox calibration curves were prepared for each assay, and linearity was verified by linear regression, with coefficients of determination (R^2^) higher than 0.99. Results were expressed as mg Trolox equivalents (TE) per 100 g of oil.

#### 2.8.1. Preparation of Methanolic Oil Extract

The methanolic oil extract was prepared by weighing 2.5 g of tumbo seed oil into a Falcon tube and adding 5 mL of 80% methanol. The mixture was vigorously vortexed for 2 min using a Vortex-Genie 2 digital vortex mixer (Scientific Industries, Bohemia, NY, USA) and then centrifuged at 3000 rpm for 10 min in a Sorvall ST 8 benchtop centrifuge (Thermo Scientific, Thermo Fisher Scientific, Waltham, MA, USA) to achieve phase separation. The upper transparent methanolic phase was carefully collected, transferred to an amber vial, and used directly for the spectrophotometric assays to avoid turbidity and lipid interference during absorbance readings.

#### 2.8.2. ABTS Assay

The ABTS radical cation decolorization was assayed according to a previously described method [11]. The ABTS was adjusted to an absorbance of 0.70 ± 0.05 at 734 nm. An aliquot of methanolic oil extract was mixed with the ABTS working solution. The reaction mix was incubated for 6 min at room temperature, and the absorbance was measured at 734 nm.

#### 2.8.3. DPPH Assay

The DPPH was performed according to a procedure described in an earlier work [10]. A DPPH solution was prepared and adjusted to an absorbance of 0.70 at 520 nm. An aliquot of the previously prepared methanolic oil extract was mixed with the DPPH solution and incubated in the dark for 30 min at room temperature. The decrease in absorbance was measured at 520 nm.

#### 2.8.4. FRAP Assay

The FRAP was assayed per a previously described protocol [12]. The FRAP reagent, containing the Fe^3+^–TPTZ complex, was prepared and added to an aliquot of the methanolic oil extract. The reaction mix was incubated for 30 min at room temperature, and the absorbance was then measured at 593 nm.

### 2.9. Total Flavonoid Content

The total flavonoid content (TFC) was estimated by applying the aluminum chloride (AlCl_3_) colorimetric method [13]. An aliquot of methanolic oil extract was mixed with an AlCl_3_ solution, and the reaction mixture was incubated at room temperature to allow complex formation. Absorbance was measured at 415 nm. TFC was calculated utilizing a quercetin standard calibration curve and expressed as mg quercetin equivalents (QE) per 100 g of oil.

### 2.10. Total Phenolic Content

The total phenolic content (TPC) was estimated using the Folin–Ciocalteu spectrophotometric method [14]. Absorbance was measured at 760 nm. TPC was calculated employing a gallic acid standard calibration curve and expressed as mg gallic acid equivalents (GAE) per 100 g of oil.

As colorimetric assays, these methods provide estimations of total phenolic- and flavonoid-related compounds and do not allow the identification of individual compounds.

### 2.11. Statistical Analysis

All determinations were performed in triplicate using the same oil sample; therefore, the replicates corresponded to analytical replicates. Data are presented as mean ± standard deviation (SD). Since this study was descriptive and exploratory in nature and was based on a single oil batch, no inferential statistical comparisons were performed. GraphPad Prism software (version 10.2.3; GraphPad Software, Boston, MA, USA) was used only for descriptive data processing, including the calculation of means and standard deviations.

## 3. Results

### 3.1. Oil Yield

A total of 88.7 g of oil was obtained from 528.75 g of dried tumbo seeds, corresponding to a pressing yield of 16.78%. This value represents the oil yield obtained under the mechanical expeller pressing conditions used in this study. However, it should not be interpreted as total extraction efficiency, since exhaustive solvent extraction to determine the total oil content of the seeds was not performed.

### 3.2. Nutritional Composition of Tumbo Fruit Fractions

The nutritional compositions of various tumbo fruit fractions, expressed on a wet and dry basis, are presented in Table 1. Marked differences were observed among the evaluated fractions. On a dry basis, the residual seed cake stood out for its high crude fiber content (54.33%) and total protein content (9.03%), which are consistent with the concentrations of structural and nitrogenous residues typical of oilseed by-products after oil extraction. The peel presented an ether-extract content (8.11%) that ranked highest among all fractions, together with a relevant crude fiber content (34.78%), indicating that this commonly discarded fraction retains a non-negligible lipid and fiber content. The seed, in turn, showed the highest nitrogen-free extract content on a dry basis (69.83%), reflecting a predominance of carbohydrate-related components, along with a comparatively low crude fiber content (16.73%) and a moderate ether-extract content (2.77%). The pulp presented the highest moisture content on a wet basis (88.53%), followed by the peel (80.31%), reflecting the high water content typical of fresh fruit tissue, whereas the seed (9.51%) and seed cake (8.64%) showed markedly lower moisture, consistent with their dried or conditioned state prior to analysis. In general, these results indicate a differential distribution of nutrients among the fruit fractions and underscore the potential for the integral utilization of tumbo, particularly the peel and seed cake, for fiber- and lipid-related by-products.

### 3.3. Mineral Composition of Tumbo Fractions

The mineral compositions of various tumbo fruit fractions are shown in Table 2, as expressed on a dry basis. The mineral contents differed among the fractions evaluated. The seed cake presented the highest nitrogen content (1.30 ± 0.09%), whereas the pulp showed the highest copper content (42.67 ± 1.00 ppm). In contrast, the peel stood out for its highest potassium (2.35 ± 0.01%), calcium (0.40%), manganese (36.00 ppm), iron (72.00 ppm), and boron (13.00 ppm) contents. The seed cake also presented the highest zinc content (24.67 ± 0.50 ppm) and a relevant iron content (65.00 ppm), whereas the seed showed relevant concentrations of iron (41.00 ppm) and zinc (18.00 ppm). These results indicate a differential distribution of minerals among the fruit fractions, highlighting the potential for the integral utilization of tumbo.

### 3.4. Fatty Acid Profile of Tumbo Seed Oil

The fatty acid profile of tumbo seed oil is summarized in Table 3. The oil exhibited a predominance of unsaturated lipids, with total unsaturated and saturated fatty acids representing 86.44% and 13.47%, respectively. The major fatty acid was linoleic acid (C18:2, ω-6), at 75.96%, followed by oleic acid (C18:1, ω-9) at 9.72% and palmitic acid (C16:0) at 8.98%. In addition, α-linolenic acid (C18:3, ω-3) was detected at 0.60%, resulting in an ω-6/ω-3 ratio of 126. These results indicate that tumbo seed oil presents a highly unsaturated fatty acid profile, one mainly characterized by its enhanced linoleic acid content.

### 3.5. Physicochemical Characteristics, In Vitro Antioxidant Capacity, and Phenolic Compounds of Tumbo Seed Oil

Table 4 presents the physicochemical characteristics, in vitro antioxidant capacity, and phenolic compound contents of tumbo seed oil. It showed low acid (0.28 ± 0.00 mg KOH/g oil) and peroxide (2.00 meq O_2_/kg oil) values, suggesting adequate physicochemical quality, a reduced degree of lipid hydrolysis, and minimal initial oxidation. In addition, the iodine value was high (135.36 ± 0.56 g I_2_/100 g oil), indicating a high degree of unsaturation, which is consistent with the polyunsaturated fatty acid-rich lipid profile observed.

Regarding AOC, tumbo seed oil showed activities of 57.38 ± 2.51 mg TE/100 g oil, 33.20 ± 1.27 mg TE/100 g oil, and 59.13 ± 0.40 mg TE/100 g oil. These results indicate relevant antioxidant activity, which is associated with free radical-scavenging capacity and reducing power.

Furthermore, the oil presented a TPC of 342.11 ± 10.63 mg GAE/100 g oil and a TFC of 307.58 ± 32.50 mg QE/100 g oil. Overall, these results suggest that the AOC of tumbo seed oil may be due to the phenolic compounds and flavonoids which contribute to its functional potential.

## 4. Discussion

The pressing yield obtained from tumbo seeds by expeller pressing was consistent with previous studies showing that oil recovery during mechanical extraction depends primarily on seed lipid content, particle size, moisture, pressure, and temperature during processing. These factors can variably affect pressing efficiency in oilseeds, with lipid levels being one of the main determinants of final yield [15]. Likewise, mechanical oil extraction studies indicate that expeller pressing is more widely used because of its simplicity and sustainability. Although yields are usually lower than those obtained by solvent extraction, because the total amount of oil contained within the matrix is not completely recovered, expeller pressing still allows the extraction of good-quality oils [16]. The pressing yield obtained in this study (16.78%) was similar to those reported for granadilla seeds (18.42%) [1] and for tarwi oil obtained via expeller pressing, with variety-dependent yields ranging between 15% and 18% [17]. Comparable results were also reported for yellow passion fruit (*Passiflora* edulis) seeds pressed under similar mechanical conditions, where oil recovery ranged from 78% to 89%, relative to the total oil content of the seed, depending on seed moisture and provenance [18]. These values reflect extraction efficiency rather than yield relative to seed weight, and are therefore not directly comparable to the present result. This distinction illustrates the discrepancies among *Passiflora* oil studies, which often arise not only from species, origin, or maturity, but also from differences in how yield is defined and calculated across the literature. Considered together, these findings confirm that oil recovery by expeller pressing varies according to species and processing conditions, and support the viability of this method as a sustainable approach for obtaining tumbo seed oil for further compositional and technological evaluation.

Regarding nutritional composition, the compositional variability observed among the different tumbo fruit fractions was consistent with reports on tropical fruits, where each part presents a distinct nutritional profile according to its structural or metabolic function [19]. Expressing the data on both wet and dry bases allowed a clearer interpretation of nutrient distribution, since wet-basis values reflect the composition of the materials as analyzed, whereas dry-basis values allow comparison of nutrient concentrations after excluding the effect of moisture. The highest moisture contents on a wet basis were observed in the pulp (88.53%) and peel (80.31%), reflecting the high water contents typical of fresh fruit tissues (Table 1). In contrast, the seed (9.51%) and seed cake (8.64%) showed markedly lower moisture contents, consistent with their dried or conditioned state prior to analysis.

On a dry basis, the residual seed cake stood out for its high crude fiber (54.33%) and total protein (9.03%) contents, a behavior consistent with oilseed by-products, in which residues from oil extraction may concentrate structural and nitrogenous components. Recent studies indicate that these by-products may be considered potential sources of dietary fiber and protein [20]. The high crude fiber content of the peel (34.78%) further supports its potential as a fiber-rich by-product, in agreement with previous reports describing passion fruit peel as a valuable source of dietary fiber for food fortification [21]. The seed, in turn, showed the highest nitrogen-free extract content on a dry basis (69.83%), reflecting a predominance of carbohydrate-related components, along with a comparatively low crude fiber content (16.73%) and a moderate ether-extract content (2.77%). This distribution diverges from that typically reported for *P. edulis*, in which the seed, rather than the peel, is consistently described as the fraction with the highest lipid content (12.3–19.6% on a dry basis), well above the peel (4.2–10.3%) and pulp (1.1–2.9%) [22]. Taken together, although the general trend of differentiated nutrient distribution among fruit fractions was consistent with previous reports on *Passiflora* species [23], the specific distribution of lipids between seed and peel in tumbo differed from that typically observed in *P. edulis*. This highlights a species-specific variability that merits further investigation and supports the potential for integral utilization of this fruit, in line with circular-economy approaches to fruit by-product valorization [24].

The mineral profile also indicated a differential distribution of macro- and microminerals among the tumbo fractions. This observation agrees with descriptions of tropical fruits, where mineral variability may be associated with the physiological function of each tissue, its nutrient storage capacity, and external factors such as cultivation conditions and soil composition [25]. Tropical fruits are important sources of minerals, although the concentrations of these minerals vary according to species and fruit fraction. On a dry basis, the peel presented the highest contents of potassium (2.35 ± 0.01%), calcium (0.40%), manganese (36.00 ppm), iron (72.00 ppm), and boron (13.00 ppm), indicating greater mineral accumulation in this fraction. As for the finding that some mineral contents in the pulp are high, this finding contrasts with reports describing tropical fruits’ pulp as comparatively lower in minerals due to its high moisture content [26]. The elevated potassium and calcium contents may be related to their roles in osmotic balance and cell turgor. The seed cake presented the highest nitrogen (1.30 ± 0.09%) and zinc (24.67 ± 0.50 ppm) contents, along with a relevant iron level (65.00 ppm), suggesting that this by-product retains nutritionally relevant minerals after oil extraction. Such differences may arise from genuine species- and cultivar-related variability, but may also be influenced by soil composition, cultivation conditions, and fruit maturity at harvest. Collectively, these results show that tumbo fractions present differentiated mineral profiles, supporting the potential for integrated fruit utilization, especially of the peel and seed cake, as complementary sources of macro- and microminerals.

The fatty acid profile revealed a predominance of polyunsaturated fatty acids, particularly linoleic acid (ω-6), confirming that tumbo seed oil follows the general lipid pattern reported across the *Passifloraceae* family, although with notable quantitative differences among species. A predominance of linoleic acid has also been reported in granadilla seed oil [1], while passion fruit seed oil has been reported to contain 60–80% unsaturated fatty acids, with linoleic acid as the predominant component [27]. In *P. edulis* obtained by expeller pressing, linoleic acid ranged from 67.0% to 69.0%, along with relevant levels of oleic acid (16.0–17.5%), palmitic acid (11.0–11.3%), and stearic acid (2.9–3.2%) [18]. A broader comparison across three *Passiflora* species (*P. edulis* Purple, *P. quadrangularis*, and *P. maliformis*) showed linoleic acid ranging from 63.20% to 70.42%, palmitic acid from 9.67% to 13.14%, stearic acid from 2.40% to 3.13%, and total unsaturated fatty acids from 82.84% to 85.69% [28], indicating that, despite genuine interspecific variability, linoleic acid consistently dominates the fatty acid profile across the genus. Similarly, *P. setacea* seed oil, a wild Brazilian species, showed linoleic acid at 64.69% and oleic acid at 20.67% [29], while *P. edulis* oil from Vietnam showed linoleic acid at 66.94% and oleic acid at 18.86% [30]. In comparison, tumbo seed oil showed a linoleic acid content (75.96%) that exceeded all previously reported values for *Passiflora* species, while its oleic acid content (9.72%) was markedly lower than those reported for *P. edulis*, *P. setacea*, *P. quadrangularis*, and *P. maliformis* (13.6–20.7%). This inverse relationship between linoleic and oleic acid contents indicates that tumbo seed oil presents an ω-6 fatty acid profile even more concentrated than those of other characterized *Passiflora* species, which may be attributed to genetic distance—tumbo belongs to a different subgenus, and is adapted to high-altitude Andean conditions, unlike the lowland-adapted *P. edulis* and *P. setacea*—as well as to environmental, maturity-related, or processing-related factors affecting desaturase enzyme activity during seed lipid biosynthesis. In contrast, oils such as olive oil present an unsaturated fatty acid profile dominated by oleic acid, which enhances oxidative stability, compared with oils rich in polyunsaturated fatty acids [31]. The low ω-3 fatty acid content of tumbo seed oil, together with a high ω-6/ω-3 ratio, is consistent with reports indicating that tropical and Andean fruit seeds are valuable sources of fatty acids but usually contain greater concentrations of ω-6, mainly linoleic acid, than ω-3, mainly α-linolenic acid [32]. However, the low ω-3 content and high ω-6/ω-3 ratio should be considered a nutritional limitation, particularly if the oil is intended for food applications; future formulation strategies may therefore require combination with ω-3-rich oils to improve the fatty acid balance.

The physicochemical indices of tumbo seed oil were consistent with those expected for a good-quality, minimally oxidized vegetable oil. The acid value (0.28 mg KOH/g oil) indicates limited triglyceride hydrolysis and, therefore, a reduced free fatty acid content, suggesting adequate raw material quality and appropriate extraction conditions. This value was well below the maximum limits established by the *Codex Alimentarius* Standard for Named Vegetable Oils (CXS 210-1999) for both refined oils (0.6 mg KOH/g oil) and cold-pressed/virgin oils (4.0 mg KOH/g oil) [33], and was also lower than the acid value reported for *P. edulis* seed oil obtained by Soxhlet extraction (1.117 mg KOH/g oil) [34]. Similarly, the peroxide value (2.00 meq O_2_/kg oil) indicates minimal primary oxidation at the time of analysis; low peroxide values are commonly associated with higher levels of oil quality and sufficient contents of natural antioxidant compounds [35,36]. This value remained below the *Codex* limits for both refined (10 meq O_2_/kg oil) and cold-pressed/virgin oils (15 meq O_2_/kg oil) [33], fell within the range reported for other *Passiflora* species (1.43–3.23 meq O_2_/kg oil) [28], and was consistent with the low or non-detectable peroxide levels typically reported for expeller-pressed passion fruit seed oil under similar pressing conditions [18], although it was lower than the value reported for *P. edulis* seed oil obtained by Soxhlet extraction (9.267 meq O_2_/kg oil) [34]. The high iodine value (135.36 g I_2_/100 g oil) reflects an increased degree of unsaturation, characteristic of vegetable oils rich in polyunsaturated fatty acids and common in oils from tropical and non-conventional oilseeds [37], and was slightly higher than values reported for other *Passiflora* seed oils (124.67–131.00 g I_2_/100 g oil for *P. edulis*, *P. quadrangularis*, and *P. maliformis* [28]; 129.25 g I_2_/100 g oil for *P. edulis* [34]), which is consistent with the comparatively higher linoleic acid content of tumbo seed oil. Taken together, these physicochemical indices indicate that tumbo seed oil obtained by expeller pressing meets recognized quality standards for edible vegetable oils and is comparable to, or in some respects superior to, previously reported oils within the *Passifloraceae* family. However, the high degree of unsaturation may also increase susceptibility to lipid oxidation, indicating the need for future oxidative stability studies under storage and processing conditions.

The in vitro antioxidant capacity, evaluated by ABTS, DPPH, and FRAP assays, indicated that tumbo seed oil contains compounds capable of participating in radical-scavenging and reducing reactions. These assays provide complementary information on antioxidant behavior; however, they should be interpreted as chemical screening methods rather than as evidence of biological antioxidant effects [38]. Comparative studies have shown that the in vitro antioxidant capacity of vegetable oils varies considerably among lipid matrices, particularly according to the presence of minor compounds and extraction conditions; in some cases, virgin oils such as sesame or rapeseed have demonstrated antioxidant capacities comparable to or higher than their refined counterparts [39]. Direct comparisons of antioxidant capacity for *Passiflora* seed oil expressed as mg TE/100 g oil remain scarce in the literature, as most previous studies have focused on defatted cake extracts or methanolic/ethanolic extracts rather than on the oil itself, underscoring the value of the present data as a baseline reference for the species.

With regard to the total phenolic content (TPC) of tumbo seed oil (342.11 mg GAE/100 g oil), it was higher than those reported for conventional vegetable oils such as olive oil, whose profiles may vary depending on cultivar, extraction conditions, and storage [40], and was more than twice as high as that previously reported for yellow passion fruit (*P. edulis* f. *flavicarpa*) seed oil obtained by Soxhlet extraction (131.4 mg GAE/100 g oil, equivalent to 1314.13 mg GAE/kg oil) [41], in which only a small proportion of the total seed phenolics content is transferred to the oil during extraction. This comparatively high value may reflect greater retention of phenolic compounds during expeller pressing relative to solvent-based extraction, since expeller pressing avoids prolonged solvent contact and the elevated temperatures that can promote phenolic degradation, although this hypothesis was not directly tested in the present study. The reported levels of phenols for other seed oils differ significantly depending on the botanical source, fruit type, extraction method, and concentration, with lower concentrations recorded in pumpkin, moringa, grape, and sesame seed oils [42]. These differences could explain the measurable in vitro antioxidant capacity observed in passionfruit seed oil; however, a correlation analysis was not performed in the present study, so the relationships between total phenol content, total flavonoid content, and in vitro antioxidant capacity should be interpreted as possible associations rather than confirmed direct relationships.

Similarly, the total flavonoid content (TFC) of tumbo seed oil (307.58 mg QE/100 g oil) was higher than values reported for oils such as soybean, grape, perilla, and chia, where these compounds are usually found at low or trace levels [43]. This variation suggests that antioxidant-related minor compounds may contribute to the in vitro antioxidant response of tumbo seed oil. In vegetable oils, the preservation of these compounds may depend on extraction and processing conditions, which are important factors for maintaining oil quality [44]. Extracts from wild *Passiflora* species (*P. setacea*, *P. alata*, and *P. tenuifila*) seed cakes obtained after oil pressing have shown considerably higher phenolic contents (approximately 600–1800 mg GAE/100 g of extract) [45], confirming that the defatted cake retains a substantial proportion of the phenolic fraction not transferred to the oil, and suggesting that the tumbo seed cake—not evaluated for phenolic content in the present study—may represent an additional source of antioxidant compounds worth characterizing in future work. Overall, these differences in TPC and TFC across studies highlight that phenolic and flavonoid retention in *Passiflora* oils and by-products depends strongly on species, extraction method, and whether the oil, extract, or press cake is being analyzed, reinforcing the need for standardized reporting when comparing antioxidant-related compounds across the genus.

The total phenolic content (TPC) and total flavonoid content (TFC) observed in tumbo seed oil suggest the presence of antioxidant-related compounds in the methanolic oil extract. However, these values should be interpreted with caution, since the Folin–Ciocalteu and aluminum chloride methods are colorimetric assays that may respond to different reducing or complex-forming compounds; TPC and TFC therefore represent global estimates rather than evidence of specific phenolic or flavonoid compounds. Further analyses using high-performance liquid chromatography (HPLC) are required to identify and quantify individual phenolic and flavonoid compounds in tumbo seed oil. In addition, other minor oil components, such as phospholipids, sterols, and individual polyphenolic compounds, were not analyzed in the present study; future work should include detailed compositional profiling to better characterize the minor bioactive fraction of tumbo seed oil.

In general, the results indicate that tumbo seed oil obtained by expeller pressing is characterized by a high proportion of unsaturated fatty acids, especially linoleic acid, low initial acidity and peroxide values, and measurable in vitro antioxidant capacity. These findings provide baseline compositional information and suggest that this oil may be considered a promising non-conventional vegetable oil for future technological and food-related studies. However, its oxidative stability, safety (including heavy metals, mycotoxins, pesticide residues, and microbial load), shelf-life behavior, detailed bioactive compound profile, and potential biological effects need to be evaluated in future studies before proposing specific functional or health-related applications.

## 5. Conclusions

Tumbo seed oil obtained by expeller pressing showed a composition characterized by a high proportion of polyunsaturated fatty acids, mainly linoleic acid, together with measurable total phenolic content, total flavonoid content, and in vitro antioxidant capacity, as evaluated by the ABTS, DPPH, and FRAP assays. These findings provide baseline information on the physicochemical, nutritional, fatty acid, and antioxidant-related characteristics of this non-conventional Andean fruit seed oil.

The high ω-6/ω-3 ratio, the absence of oxidative stability assays, and the use of colorimetric methods for total phenolic content and total flavonoid content should be considered limitations of the present study. Therefore, further research is required to evaluate oxidative stability, shelf-life behavior, and safety, in addition to the determination of detailed bioactive compound profiles using chromatographic techniques and potential biological effects in appropriate experimental models, before proposing specific functional or health-related applications.

## Figures and Tables

**Table 1:** Nutritional composition of the different tumbo fruit fractions on a wet and dry basis.

Component (%)	Peel WB	Peel DB	Pulp WB	Pulp DB	Seed WB	Seed DB	Seed Cake WB	Seed Cake DB
Moisture	80.31 ± 0.04	—	88.53 ± 0.02	—	9.51 ± 0.19	—	8.64 ± 0.09	—
Total protein (%N × 6.25)	1.47 ± 0.02	7.45 ± 0.13	0.74 ± 0.02	6.45 ± 0.16	5.95 ± 0.10	6.57 ± 0.12	8.25 ± 0.10	9.03 ± 0.11
Ether extract	1.60 ± 0.03	8.11 ± 0.15	0.13 ± 0.01	1.16 ± 0.05	2.50 ± 0.09	2.77 ± 0.10	3.95 ± 0.06	4.32 ± 0.06
Crude fiber	6.85 ± 0.16	34.78 ± 0.73	2.28 ± 0.04	19.88 ± 0.30	15.14 ± 0.45	16.73 ± 0.47	49.63 ± 0.20	54.33 ± 0.17
Ash	0.58 ± 0.03	2.96 ± 0.13	0.73 ± 0.01	6.40 ± 0.05	3.70 ± 0.02	4.09 ± 0.01	1.51 ± 0.04	1.65 ± 0.04
Nitrogen-free extract	9.20 ± 0.14	46.70 ± 0.79	7.58 ± 0.05	66.10 ± 0.40	63.19 ± 0.43	69.83 ± 0.51	28.02 ± 0.07	30.67 ± 0.08

Note: Values are expressed as mean ± SD. WB: wet basis; DB: dry basis. Moisture is not applicable on a dry basis because DB values are calculated after moisture removal. Ether extract refers to the lipid fraction determined by solvent extraction. Nitrogen-free extract was calculated by difference.

**Table 2:** Macro- and micromineral contents of tumbo fractions on a dry basis.

Component	Unit	Seed	Pulp	Peel	Seed Cake
Nitrogen	%	1.12 ± 0.01	1.06 ± 0.10	0.85 ± 0.01	1.30 ± 0.09
Phosphorus	%	0.16 ± —	0.12 ± —	0.17 ± —	0.17 ± 0.01
Potassium	%	1.23 ± —	1.30 ± 0.05	2.35 ± 0.01	0.50 ± 0.03
Calcium	%	0.05 ± —	0.22 ± 0.01	0.40 ± —	0.03 ± —
Magnesium	%	0.09 ± —	0.11 ± —	0.11 ± —	0.11 ± —
Sulfur	%	0.07 ± —	0.13 ± 0.01	0.12 ± —	0.04 ± 0.03
Sodium	%	0.01 ± —	0.09 ± —	0.06 ± —	0.01 ± 0.01
Copper	ppm	8.00 ± —	42.67 ± 1.00	2.00 ± —	38.33 ± 0.50
Manganese	ppm	8.00 ± —	8.00 ± 0.87	36.00 ± —	8.67 ± 0.50
Iron	ppm	41.00 ± —	34.67 ± 3.28	72.00 ± —	65.00 ± —
Zinc	ppm	18.00 ± —	18.33 ± 0.50	10.00 ± —	24.67 ± 0.50
Boron	ppm	7.00 ± —	12.67 ± 1.00	13.00 ± —	5.67 ± 1.00

Note: Values are expressed as mean ± SD of three analytical replicates. Values were reported using two decimal places; SD values shown as “—” indicate variation below the displayed decimal precision.

**Table 3:** Fatty acid profile of tumbo seed oil.

Code	Fatty Acid (FA)	%
C12:0	Lauric acid	0.15 ± 0.01
C14:0	Myristic acid	0.40 ± 0.02
C16:0	Palmitic acid	8.98 ± 0.44
C16:1	Palmitoleic acid	0.13 ± 0.01
C18:0	Stearic acid	3.56 ± 0.20
C18:1n-9	cis-9 Oleic acid (ω-9)	9.72 ± 0.12
C18:2n-6	Linoleic acid (ω-6)	75.96 ± 0.76
C20:0	Arachidic acid	0.26 ± 0.01
C20:1	cis-11-Eicosenoic acid	0.08 ± —
C18:3n-3	α-Linolenic acid (ω-3)	0.60 ± 0.02
C20:2	cis-11,14-Eicosadienoic acid	0.05 ± —
C22:0	Behenic acid	0.12 ± 0.01
	Total saturated fatty acids (SFA)	13.47 ± 0.63
	Total unsaturated fatty acids (UFA)	86.44 ± 0.63
	SFA/UFA ratio	0.16 ± 0.01
	ω-6/ω-3 ratio	126.00 ± 5.17

Note: Values are expressed as relative percentages of the total fatty acids detected. SFA: saturated fatty acids; UFA: unsaturated fatty acids. Values are expressed as mean ± SD of three analytical replicates and rounded and reported using two decimal places; values shown as “—” indicate variation below the displayed decimal precision.

**Table 4:** Physicochemical characteristics, in vitro antioxidant capacity, and phenolic profile of tumbo seed oil.

Measurement	Value
Physicochemical characteristics	
Acid value, mg KOH/g oil	0.28 ± 0.00
Iodine value, g I_2_/100 g oil	135.36 ± 0.56
Peroxide value, meq O_2_/kg oil	2.00 ± 0.00
Antioxidant capacity	
ABTS, mg TE/100 g oil	57.38 ± 2.51
DPPH, mg TE/100 g oil	33.20 ± 1.27
FRAP, mg TE/100 g oil	59.13 ± 0.40
Total phenolic and flavonoid contents	
Total phenolic content, mg GAE/100 g oil	342.11 ± 10.63
Total flavonoid content, mg QE/100 g oil	307.58 ± 32.50

Note: Values are expressed as mean ± standard deviation (*n* = 3). TE: Trolox equivalents; GAE: gallic acid equivalents; QE: quercetin equivalents; ABTS: 2,2′-azino-bis (3-ethylbenzothiazoline-6-sulfonic acid); DPPH: 2,2-diphenyl-1-picrylhydrazyl; FRAP: ferric reducing antioxidant power. Values were reported using two decimal places.

## Data Availability

The original contributions presented in this study are included in the article. Further inquiries can be directed to the corresponding author.

## Abbreviations

The following abbreviations are used in this manuscript:

Abbreviation	Meaning
AAS	Atomic absorption spectrophotometry
ABTS	2,2′-azino-bis(3-ethylbenzothiazoline-6-sulfonic acid)
DPPH	2,2-diphenyl-1-picrylhydrazyl
FRAP	Ferric reducing antioxidant power
TE	Trolox equivalents
GAE	Gallic acid equivalents
QE	Quercetin equivalents
WB	Wet basis
DB	Dry basis
NFE	Nitrogen-free extract
SFA	Saturated fatty acids
UFA	Unsaturated fatty acids
PUFA	Polyunsaturated fatty acids
MUFA	Monounsaturated fatty acids
FAME	Fatty acid methyl esters
GC-MS/MS	Gas chromatography–tandem mass spectrometry
TPC	Total phenol content
TFC	Total flavonoid content
AOCS	American Oil Chemists’ Society
AOAC	Association of Official Analytical Chemists
